# Establishment of the Medical Department of Niagara University1Read at the seventy-fifth anniversary of the Medical Society of the County of Erie, January 14, 1896.

**Published:** 1896-05

**Authors:** John Cronyn

**Affiliations:** President of the faculty; 55 West Swan Street


### ESTABLISHMENT OF THE MEDICAL DEPARTMENT OF NIAGARA UNIVERSITY.[1]

By JOHN CRONYN, M. D., president of the faculty.

THE religious order to which Niagara University owes its existence was founded about 270 years ago by St. Vincent de Paul, who was also founder of the community distinctively known as the "Sisters of Charity." In 1856, Father Lynch, C. M., —subsequently Archbishop of Toronto—established, near Suspension Bridge, on historic ground overlooking the Niagara, the College and Seminary of our Lady of Angels, placing it in charge of the Vincentians, of which order he was himself a member. The schools began then a work that has been steadily and increasingly prosperous.

The subject of adding other departments to the colleges of theology and arts was at several times considered. Indeed, with Father Rice, of revered memory—second president of the institution—long ago, I had many conferences in regard to the possible

1. Read at the seventy-fifth anniversary of the Medical Society of the County of Erie, January 14, 1896.

and much-needed medical school. Not, however, until 1883 were some of these plans realised. In that year the colleges were erected into a university by the Regents of the University of the State of New York. Invested with the full powers and authority of a university, Niagara acquired also, by an act of the legislature, the right to establish colleges and grant degrees in both Erie and Niagara counties. In 1883, the medical faculty was organised, which began teaching in September of that year.

Truth compels me to add that Erie County Society served Niagara University and the new medical school only by opposing the granting of its charter to the first and by hindering as much as possible the establishment of the second. We have forgiven the society.

Although the trustees were anxious and prepared to organise the medical department, so far as its *personnel* and authority to teach might go, all financial responsibility devolved upon the men who composed the faculty. They put their hands into their pockets and spent the to them not inconsiderable sum of $50,000, which represented in some degree their earnestness of purpose and the sacrifices they were ready to make for the cause of higher medical education. This, in brief, was the beginning of the Medical Department of Niagara University, of which you have done me the honor to ask me to speak.

The college opened in 1883 with a class of ten young men. Dr. Lothrop, Doctors Gay, Tremaine, Heath, Ingraham, Hubbell, Davidson, Campbell, Pitt, Stockton, Mr. Congdon, S. Tucker Clark and myself comprised the faculty.

Very early in its history death robbed the faculty of several valued members. Other causes made changes more or less inevitable, but the most active workers of the initial year are still active. They have seen the faculty grow to the number of sixteen professors and thirteen adjunct professors and lecturers, the students multiply tenfold, the Sisters' Hospital and other institutions, whose wards are open for Niagara's clinics, prosper and grow in a notable manner.

In conclusion, let me say that the Medical Department of Niagara University has many reasons for existing and for gladness in its existence, not the least being the fact that it has insisted upon a three years' course of study as the minimum ; that it has obliged its students to preliminary examinations in Latin and higher English, or to give evidence that they have made the necessary studies therein; that it has compelled other colleges in the state to follow its example; that it has been instrumental in obliging the state authorities to enact severer laws in regard to the qualifications of medical men; and finally, that, largely in consequence of its action, the profession all over the state and the people in general have been benefited beyond, far beyond, what it is now possible to realise.

55 West Swan Street.

